# Cross-Subject EEG Emotion Recognition With Self-Organized Graph Neural Network

**DOI:** 10.3389/fnins.2021.611653

**Published:** 2021-06-09

**Authors:** Jingcong Li, Shuqi Li, Jiahui Pan, Fei Wang

**Affiliations:** ^1^School of Software, South China Normal University, Guangzhou, China; ^2^Pazhou Lab, Guangzhou, China; ^3^School of Computer Science, South China Normal University, Guangzhou, China

**Keywords:** SEED dataset, graph neural network, cross-subject, emotion recognition, graph construction

## Abstract

As a physiological process and high-level cognitive behavior, emotion is an important subarea in neuroscience research. Emotion recognition across subjects based on brain signals has attracted much attention. Due to individual differences across subjects and the low signal-to-noise ratio of EEG signals, the performance of conventional emotion recognition methods is relatively poor. In this paper, we propose a self-organized graph neural network (SOGNN) for cross-subject EEG emotion recognition. Unlike the previous studies based on pre-constructed and fixed graph structure, the graph structure of SOGNN are dynamically constructed by self-organized module for each signal. To evaluate the cross-subject EEG emotion recognition performance of our model, leave-one-subject-out experiments are conducted on two public emotion recognition datasets, SEED and SEED-IV. The SOGNN is able to achieve state-of-the-art emotion recognition performance. Moreover, we investigated the performance variances of the models with different graph construction techniques or features in different frequency bands. Furthermore, we visualized the graph structure learned by the proposed model and found that part of the structure coincided with previous neuroscience research. The experiments demonstrated the effectiveness of the proposed model for cross-subject EEG emotion recognition.

## 1. Introduction

Human emotion is a complex psychophysiological process that plays an important role in daily communications. Emotion recognition is a significant and fundamental research topic in affective computing and neuroscience (Cowie et al., 2001). In general, human emotions can be recognized using data from different modalities, such as facial expression images, body language, textual information and physiological signals such as electromyogram (EMG), electrocardiogram (ECG), and electroencephalogram (EEG) (Busso et al., 2004; Shu et al., 2018). EEG is a widely used technique in neuroscience research that is able to directly capture brain signals that could reflect neural activities in real time. Therefore, EEG-based emotion recognition has received considerable attention in the areas of affective computing and neuroscience (Coan and Allen, 2004; Lin et al., 2010; Alarcao and Fonseca, 2017; Li et al., 2019).

In order to facilitate EEG-based emotion recognition research, the SJTU emotion EEG dataset (SEED) was released (Duan et al., 2013). In addition, its evolutionary dataset termed SEED-IV was also available (Zheng et al., 2018). Before the experiments on SEED and SEED-IV datasets, a series of film clips with different emotional tendencies were chosen as stimulation materials. In the SEED dataset, happy, sad and neutral emotions were included, while the SEED-IV dataset consisted of happy, sad, fear and neutral emotions. During the experiments, each participant watched the film clips while his/her EEG signals were recorded with a 62-channel ESI NeuroScan System. Consequently, the recorded EEG signals and the corresponding emotion labels of film clips can be used to train an emotion recognition model. If the trained emotion recognition model is effective, we will be able to decode the emotions of a new participant when he/she watched a film. Therefore, based on the SEED and SEED-IV datasets, different emotion recognition methods can be evaluated on common benchmarks.

In the past few years, many feature extraction and machine learning approaches have been proposed for EEG-based emotion recognition. In an original research on SEED dataset, the features of the energy spectrum (ES), differential entropy (DE), rational asymmetry (RASM), and differential asymmetry (DASM) were proven to be effective features for EEG-based emotion recognition (Duan et al., 2013). To explore different EEG features for cross-subject emotion recognition, 18 kinds of linear and non-linear EEG features were evaluated (Li et al., 2018b). Moreover, a machine learning technique was used to investigate stable EEG patterns for emotion recognition and achieved high performance on SEED and DEAP emotion recognition datasets (Zheng et al., 2017). To eliminate the individual differences in EEG signals, a deep adaption network (DAN) was proposed and applied on the SEED and SEED-IV datasets to conduct cross-subject emotion recognition (Li et al., 2018a). A novel group sparse canonical correlation analysis (GSCCA) method was proposed for simultaneous EEG channel selection and emotion recognition (Zheng, 2016).

Recently, deep learning and graph representation methodology were proven to be powerful tools to model structured data and achieved significant performance in many applications (Linial et al., 1995; Even, 2011). A deep belief network (DBN) was applied to process differential entropy features extracted from multichannel EEG signals (Zheng et al., 2014). To investigate critical frequency bands and channels for EEG-based emotion recognition, a deep neural network was proposed (Zheng and Lu, 2015). Long-short term memory (LSTM) was used to learn features from EEG signals, and these features were discriminative for emotion recognition on the DEAP dataset (Alhagry et al., 2017). EEG signals were recorded by EEG caps placed on the scalp, and these data can be considered to be a typical kind of structured data (Micheloyannis et al., 2006). Accordingly, graph representation approaches also achieved impressive performance in handling EEG signals in emotion recognition experiments. For example, a dynamic graph convolutional neural network (DGCNN) was proposed for emotion recognition, and its graph structure was determined by a dynamic adjacency matrix that reflected the intrinsic relationships between different EEG electrodes (Song et al., 2019b). In order to explore the deeper-level information of graph-structured EEG data, a graph convolutional broad network (GCB-net) was proposed and achieved high performance on the SEED and DREAMER datasets (Zhang et al., 2019). To capture both local and global interchannel relations, a regularized graph neural network (RGNN) was proposed and achieved state-of-the-art performance on the SEED and SEED-IV datasets (Zhong et al., 2020).

In this paper, we proposed a novel model for cross-subject EEG emotion recognition and evaluated the model on two common datasets. The main contributions of this paper can be summarized as follows:

1. A novel cross-subject emotion recognition model, termed the self-organized graph neural network (SOGNN), was proposed.
2. The SOGNN is able to achieve state-of-the-art emotion recognition performance with cross-subject accuracy of 86.81% on the SEED dataset and 75.27% on the SEED-IV dataset.
3. Interchannel connections and time-frequency features are aggregated by the self-organized graph construction module, graph convolution and hierarchical structure of the SOGNN to improve the cross-subject emotion recognition performance.

The remainder of this paper is organized as follows. The EEG emotion recognition datasets (SEED and SEED-IV) and the proposed SOGNN model are presented in section 2. In section 3, numerical emotion recognition experiments are conducted. In addition, the performance of the current methods and the proposed methods are presented and compared. Some discussions and analysis of the proposed model are presented in section 4. The conclusions of this paper are given in section 5.

## 2. Materials and Methods

### 2.1. EEG Emotion Recognition Datasets

In order to facilitate EEG-based emotion recognition research, the SJTU emotion EEG dataset (SEED) was released on http://bcmi.sjtu.edu.cn/~seed/ (Duan et al., 2013). In addition, its evolutionary dataset termed SEED-IV was also available (Zheng et al., 2018). Before the experiments on the SEED and SEED-IV datasets, a series of film clips with different emotional tendencies were chosen as stimulation materials. The SEED dataset includes happy, sad and neutral film clips while the SEED-IV dataset consists of happy, sad, fear and neutral film clips. During the experiments, each participant watched film clips while his/her EEG signals were recorded with a 62-channel ESI NeuroScan System.

In the SEED and SEED-IV datasets, 15 subjects (7 males and 8 females) participated in the experiments. During the experiments, 62-channel EEG signals of each subject were recorded when he/she was watching film clips with different emotion labels. There are 675 EEG samples (45 samples * 15 subjects) in SEED datasets. For each subject, there are 15 samples of happy, 15 samples of sad, and 15 samples of neutral emotion. There are 1,080 samples (72 samples * 15 subjects) in SEED-IV dataset. For each subject, there are 4 different kinds of emotion including happy, sad, fear and neutral emotion that the number of each emotion class is 18. So the number of samples per subject/class are balanced.

The signals were synchronously recorded at a 1,000 Hz sampling rate. Bandpass frequency filters of 0–75 and 1–75 Hz were applied to filter the unrelated artifacts for the SEED and SEED-IV datasets, respectively. To accelerate the computation, the signals were downsampled with sampling frequency of 200 Hz. In addition, the dataset provider applied the linear dynamic system approach to filter out noise and artifacts that were unrelated to the EEG features (Shi and Lu, 2010; Zheng et al., 2018). In the two datasets, the EEG features of the differential entropy (DE), power spectral density (PSD), asymmetry(ASM), differential asymmetry (DASM), differential caudality (DCAU), and radial asymmetry (RASM) were provided. The DE feature and PSD feature extract contents about the frequency and energy spectrum, respectively; the DASM feature and RASM feature obtain asymmetrical information of EEG channels, and DCAU feature computes the differences between channel pairs. Compared with the other features, the DE feature is more discriminative for emotion recognition according to the previous research (Duan et al., 2013; Song et al., 2019b; Zhong et al., 2020).

Therefore, we used DE features as the input data for our model. The DE features are frequency domain features that are calculated by a 512-point short-time Fourier transform with a non-overlapped Hanning window of 1 s and averaged in 5 frequency bands, e.g., δ band (1–3 Hz), θ band (4–7 Hz), α band (8–13 Hz), β band (14–30 Hz), and γ band (31–50 Hz). As a result, the output DE feature can be represented as a 5 × *T* matrix in which *T* denotes the time window which is dependent on the stimulated film clip. The time window *T* of the SEED dataset ranges from 185 to 265 while the window of SEED-IV ranges from 12 to 64. For normalization, the features with a short time window will be zero-padded to a length of 265 for SEED dataset and a length of 64 for the SEED-IV dataset.

Based on the benchmark SEED and SEED IV datasets, different EEG emotion recognition models can be evaluated and compared with each other.

### 2.2. Self-Organized Graph Neural Network

Generally, EEG signal can be considered to be a typical kind of structured data and defined on a graph (Micheloyannis et al., 2006). Graph representation techniques and graph neural networks were proven to be effective in processing brain signals (Petrosian et al., 2000; de Haan et al., 2009; Varatharajah et al., 2017; Zhang et al., 2020). Here, the EEG signal is defined on a graph model as follows:

(1) $$\begin{array}{l} \begin{array}{c} G=\left(V,E,A\right)\\ V=\left\{v_{i}|i=1,\ldots ,N\right\}\\ E=\left\{e_{ij}|v_{i},v_{j}\in V\right\}\\ A=\left\{a_{ij}\right\} \end{array} \end{array}$$

where V denotes the nodes (a total of *N* nodes) in graph G, E are the connected edges between different nodes, each node denotes one EEG electrode, *A* ∈ ℝ^*N*×*N*^ is the adjacency matrix, and its element *a*_*ij*_ denotes the adjacent connection weight between nodes *v*_*i*_ and *v*_*j*_. Consequently, the structure of a graph is determined by its adjacency matrix.

**Figure 1:**
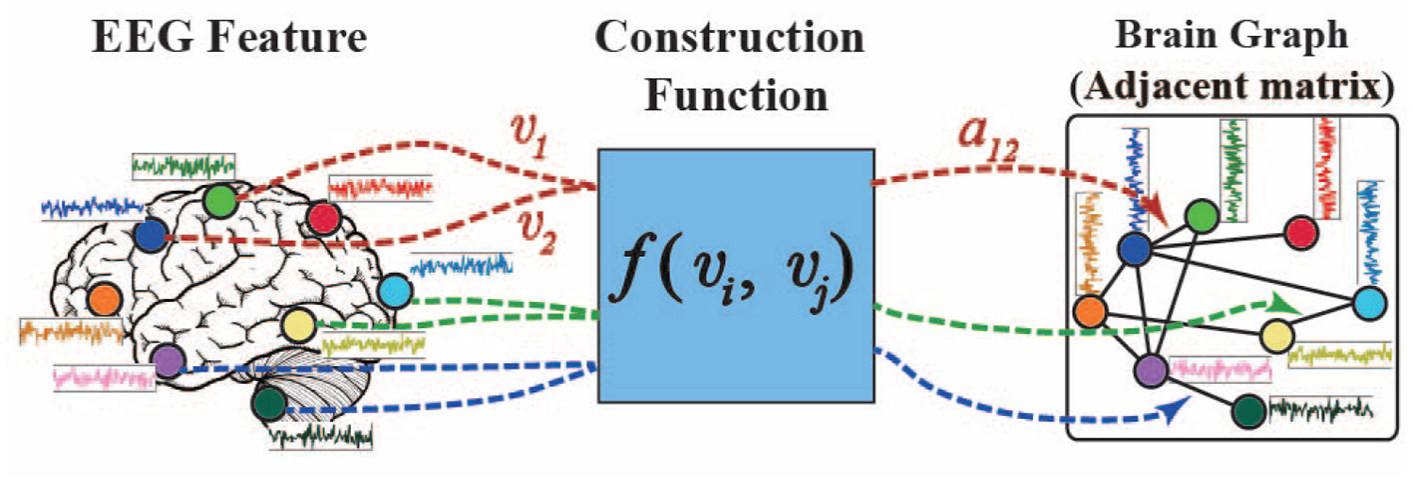
General brain graph construction function.

As shown in Figure 1, the brain graph structure is predefined by a distance function *f* between different channels in many previous studies (Micheloyannis et al., 2006; Ktena et al., 2018; Wang et al., 2018; Zhang et al., 2019; Zhong et al., 2020). However, the predefined and fixed graph structures could not properly model the dynamic brain signals of different subjects in different emotion states.

Here, we propose a self-organized graph construction module for modeling EEG emotion features. The proposed self-organized graph is determined by the input brain signals rather than based on a predefined graph structure as in many previous researches. The adjacent weight *a*_*ij*_ of the self-organized graph is defined by function *f*(*v*_*i*_, *v*_*j*_) as

(2) $$\begin{array}{l} a_{ij}=f\left(v_{i},v_{j}\right)=\frac{\exp\left(\theta \left(v_{i}W\right)\theta {\left(v_{j}W\right)}^{T}\right)}{\sum_{i=1}^{N}\exp\left(\theta \left(v_{i}W\right)\theta {\left(v_{j}W\right)}^{T}\right)} \end{array}$$

where *v* ∈ ℝ^1×*F*^ is a feature vector of one node (i.e., EEG electrode) in $V\in {\mathbb{R}}^{N\times F}$, there are a total of *N* nodes (EEG electrodes), *W* ∈ ℝ^*F*×*L*^ and θ are the weight and tanh activation function of a linear layer, respectively; and the exponential function is part of the softmax activation function for normalization and obtains a positive and bounded adjacent weight. The linear layer work as a bottleneck to reduce computational cost.

**Figure 2:**
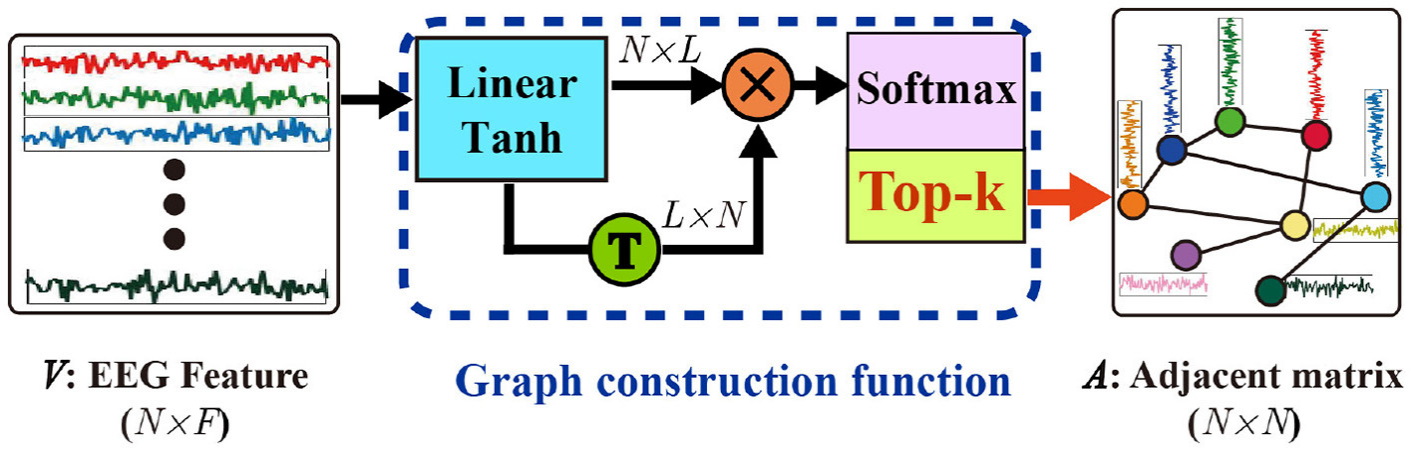
Self-organized graph construction module.

To clarify the details of the self-organized graph construction module, we also presented its matrix operation form in Figure 2. The self-organized adjacent matrix can be calculated as follows:

(3) $$\begin{array}{l} G=Tanh\left(VW\right) \end{array}$$

(4) $$\begin{array}{l} A=Softmax\left(GG^{T}\right) \end{array}$$

where $V\in {\mathbb{R}}^{N\times F}$ is the input EEG features whose row vectors are node features of the graph to build, the *W* ∈ ℝ^*F*×*L*^ denote the weight of a linear layer, we adopted tanh activation function, *G* ∈ ℝ^*N*×*L*^ is the output of the linear layer, softmax activation function is applied to obtain a positive and bounded adjacent matrix *A*. With the self-organized graph construction module, the graph structure, is dynamically constructed by the corresponding input features.

Generally, the computational costs of sparse graphs are much lower than those of dense graphs. To construct a sparse graph, we adopt a top-k technique in which only the k largest weights of the adjacent matrix will be maintained while the small connection weights will be set to zero. The top-k operation is applied as follow

(5) $$\left\{ \begin{array}{l} for\;i=1,2,\cdots ,N\\ \;index=argtopk\left(A[i,:]\right)\\ \;A[i,\bar{index}]=0 \end{array}\right.$$

where *argtopk*(·) is a function to obtain the index of the top-k largest values of each vector *A*[*i*, :] in adjacent matrix *A*, and $\bar{index}$ denotes the index of those values that do not belong to the top-k largest values in *A*[*i*, :]. As a result, only the k largest values in each row vector of adjacent matrix *A* are maintained while the remaining values will be set to zero. Actually, the top-k strategy can be considered as a modified max-pooling layer. Therefore, the parameters of the network can be updated as the network with max-pooling layers with backpropagation.

With the self-organized graph construction module, the graph structure is dynamically constructed by the corresponding input EEG features. Then, the newly built graphs can be processed by the graph convolutional layers to extract the local/global connection features for emotion recognition.

**Figure 3:**
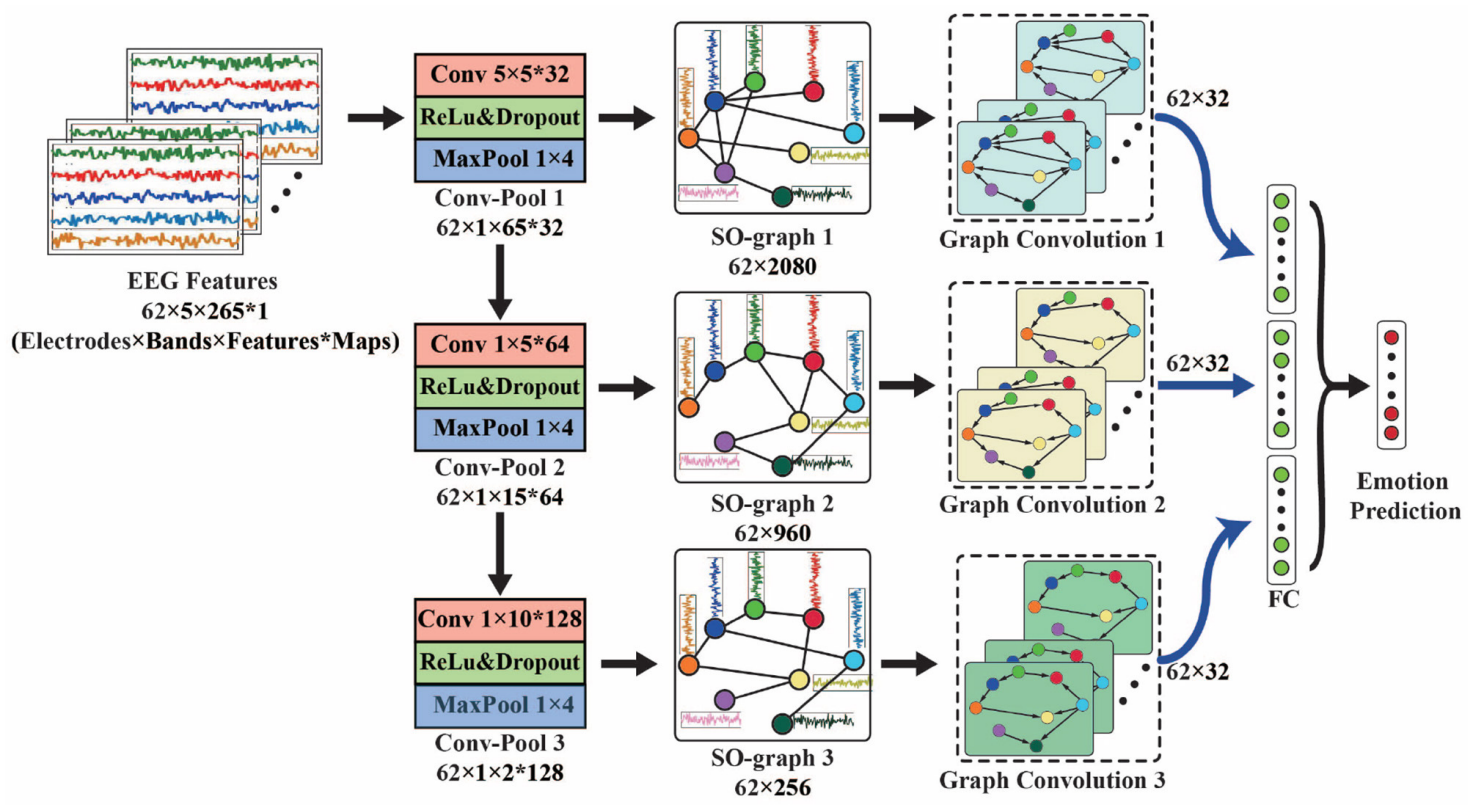
Self-organized graph neural network for EEG emotion prediction.

Based on the self-organized graph construction module, we propose SOGNN as shown in Figure 3. The SOGNN is composed of three conv-pool blocks, three self-organized graph layers, three graph convolution layers, one fully-connected layer and an output layer.

For the proposed SOGNN model, its input EEG feature is sized *Electrodes* × *Bands* × *TimeFrames*. To simplify the illustration of the model, we take the SEED dataset with a 62 × 5 × 265 input feature as an example in Figure 3. *Maps indicates the number of output feature maps of each layer. In each conv-pool block, standard convolution and max-pooling layers were applied to extract features for each EEG electrode independently. Therefore, the features of different EEG electrodes will not mix with each other so that the corresponding graph structure can be maintained. In the conv-pool 1 block, the 5 × 5 convolutional kernel extracts features in a window of 5 frequency bands and 5 time frames. Therefore, the output features are sized 62 × 1 × 261 in the SEED dataset. A 1 × 4 max-pooling layer is applied to downsample the features of the SEED dataset. Then the output feature map of conv-pool 1 block is sized 62 × 1 × 65 for the input feature of SEED. For the SEED-IV dataset, 1 × 2 max-pooling layers are used. A convolutional kernel 1 × 5 was applied in conv-pool 2 and 3. There are 32, 64, and 128 convolutional kernels in conv-pool 1-3 blocks that will obtain 32, 64, and 128 output feature maps, respectively.

The output of each conv-pool block was reshaped as a matrix with the shape of electrodes × features and fed into self-organized graph layers (SO-graphs 1-3). In the SO-graph layer, the feature of each EEG electrode remains unchanged, and only the adjacent weights between different EEG electrodes are calculated according to (2)–(5). For each SO-graph layer, there are 64 linear units, 32 output units and top-10 adjacent weights. With different input features, the graph features of the SO-graph 1-3 layers are different. Next, we applied graph convolution layers to process these graph features.

According to previous research (Bruna et al., 2013; Song et al., 2019b), spectral graph convolution multiplied a signal *x* ∈ ℝ^*n*^ with a graph convolution kernel Θ by a graph convolution operator ${*}_{G}$ as,

(6) $$\begin{array}{l} \Theta {*}_{G}x=\Theta \left(L\right)x=\Theta \left(U\Lambda U^{T}\right)x=U\Theta \left(\Lambda \right)U^{T}x \end{array}$$

where graph Fourier basis *U* ∈ ℝ^*N*×*N*^ is the matrix of eigenvectors for the normalized graph Laplacian $L=I_{n}-D^{-1/2}AD^{-1/2}=ULU^{T}\in {\mathbb{R}}^{N\times N}$ (*I*_*n*_ is an identity matrix, *D* ∈ ℝ^*N*×*N*^ is the diagonal degree matrix with $D_{ii}=\underset{j}{\sum }A_{ij}$, *A* ∈ ℝ^*N*×*N*^ is the adjacency matrix mentioned in Equation 1); Λ ∈ ℝ^*N*×*N*^ is the diagonal matrix of the eigenvalues of *L*, and filter Θ(Λ) is also a diagonal matrix. According to this definition, a graph signal *x* is filtered by a kernel Θ with multiplication between Θ and graph Fourier transform *U*^*T*^*x* (Shuman et al., 2013).

The outputs of the graph convolution layers were flattened and concatenated as a feature vector. This feature vector will be fed into fully-connected (FC) layer with a softmax activation function to predict emotional states. The proposed SOGNN model can be trained by minimizing the cross-entropy error of its prediction and ground truth. As a result, the loss function is defined as

(7) $$\begin{array}{l} L=-\underset{i\in \Omega }{\sum }\underset{c}{\sum }y_{ic}\log\left(p_{ic}\right)+\left(1-y_{ic}\right)\log\left(1-p_{ic}\right) \end{array}$$

where *p*_*ic*_ is the output value of the *c*-th output unit of the SOGNN model with the input of the *i*-th training sample, *p*_*ic*_ can be considered as the model's predicted probability of the *c*-th class, *y*_*ic*_ is the ground truth, and Ω denotes all of the training samples.

## 3. Results

In this section, a series of experiments will be conducted to evaluate the proposed model. In addition, the corresponding experimental results of our method will be presented and compared with the results of the other methods. The model implementation will be publicly available at https://github.com/tailofcat/SOGNN. In our experiments, the hardware and software configuration of our system is a platform with an Nvidia Titan Xp, Ubuntu 16.04, PyTorch 1.5.1, and PyTorch-geometric 1.5.0 (Fey and Lenssen, 2019).

In order to investigate the cross-subject emotion recognition performance, a leave-one-subject-out (LOSO) cross-validation strategy was applied in the experiments. In each run of the LOSO experiment, the DE features of 14 subjects in SEED/SEED-IV are used as the training dataset while the data of the remaining subject is the validation dataset. Regarding normalization, the features of each subject will be normalized by subtracting its mean and then dividing by its standard deviation.

In order to train the proposed SOGNN model, the Adam optimizer is applied to minimize the model's loss. The proposed model was trained by the Adam optimizer with a learning rate of 0.00001, a weight-decay rate of 0.0001 and mini-batch size of 16. A drop-out operation with a dropout rate of 0.1 was applied in the training procedure to randomly block the output units of the internal layers. During the training procedure, we monitored the model's mean area under the curve (AUC) from the receiver operating characteristic curve for all emotion classes. Once the training averaged AUC score reached 0.99, the training procedure was stopped. Finally, the trained SOGNN model could be applied for emotion prediction. Once the proposed SOGNN model was trained, it could be applied to the validation dataset. For the SEED/SEED-IV database with 15 subjects, the LOSO experiment will be conducted in 15 runs. Then, the average validation accuracy can be considered as the model's performance, which can be compared with the results of other EEG-based emotion recognition models.

As shown in Table 1, the experimental results of the proposed SOGNN and many other methods on the SEED and SEED-IV databases are presented. The bold values indicated the largest values in all methods. In the experiments of the model for one-band features, we changed the input features from 5 bands to 1 band, changed the input size of the model to fit the inputs, and retrained the model for evaluation of sub-band features. The proposed SOGNN with delta or theta band features achieved higher accuracies than the other methods with the same features. Regarding the features of the other bands, the proposed SOGNN achieved relatively high performance which was quite close to the best performing methods.

**Table 1:** Leave-one-subject-out emotion recognition accuracy (mean/standard deviation) on SEED and SEED-IV.

	**SEED**						**SEED-IV**
**Model**	**Delta band**	**Theta band**	**Alpha band**	**Beta band**	**Gamma band**	**All bands**	**All bands**
SVM (Zhong et al., 2020)	43.06/8.27	40.07/6.50	43.97/10.89	48.64/10.29	51.59/11.83	56.73/16.29	37.99/12.52
TCA (Pan et al., 2011)	44.10/8.22	41.26/9.21	42.93/14.33	43.93/10.06	48.43/9.73	63.64/14.88	56.56/13.77
SA (Fernando et al., 2013)	54.23/7.47	50.60/8.31	55.06/10.60	56.72/10.78	64.47/14.96	69.00/10.89	64.44/9.46
T-SVM (Collobert et al., 2006)	-	-	-	-	-	72.53/14.00	-
TPT (Sangineto et al., 2014)	-	-	-	-	-	76.31/15.89	-
DGCNN (Song et al., 2019b)	49.79/10.94	46.36/12.06	48.29/12.28	56.15/14.01	54.87/17.53	79.95/9.02	52.82/9.23
A-LSTM (Song et al., 2019a)	-	-	-	-	-	-	55.03/9.28
DAN (Li et al., 2018a)	-	-	-	-	-	83.81/8.56	58.87/8.13
BiDANN-S (Li et al., 2018c)	63.01/7.49	63.22/7.52	63.50/9.50	73.59/9.12	73.72/8.67	84.14/6.87	65.59/10.39
BiHDM (Li et al., 2020)	-	-	-	-	-	85.40/7.53	69.03/8.66
RGNN (Zhong et al., 2020)	64.88/6.87	60.69/5.79	60.84/7.57	**74.96/8.94**	**77.50/8.10**	85.30/6.72	73.84/8.02
SOGNN (Ours)	**70.37/7.68**	**76.00/6.92**	**66.22/11.52**	72.54/8.97	71.70/8.03	**86.81/5.79**	**75.27/8.19**

With the features of all bands, the SOGNN achieved averaged accuracy of 86.81% on the SEED dataset and 75.27% on the SEED-IV dataset, which are higher than the performances of the state-of-the-art methods, i. e. the BiHDM (Li et al., 2020) and RGNN (Zhong et al., 2020) models. The proposed SOGNN achieved a macro-F1 score of 0.8669 and an AUC score of 0.9685 on the SEED dataset. The F1 scores of happy, sad and neutral emotion class are 0.8556, 0.8577, and 0.8874. For SEED-IV dataset, it achieved a macro-F1 score of 0.7547 and an AUC score of 0.9162. The F1 scores of happy, sad, fear and neutral class are 0.7517, 0.7419, 0.7441, and 0.7810. As a typical kind of neural network, the performance of the SOGNN may be different when the model is randomly initialized by different random seeds. According to our experiments, the averaged accuracy on SEED dataset is from 0.83 to 0.88 while the averaged accuracy on SEED-IV dataset is between 0.70 and 0.78. In Table 1, we presented the medium results of the two datasets. The performance of the proposed SOGNN demonstrated its effectiveness in cross-subject emotion recognition.

Many previous graph models like DGCNN and BIDANN were based on predefined graph structure according to prior knowledge of EEG emotion signals. However, the predefined and fixed graph structures could not properly model the dynamic brain signals of different subjects in different emotion states. The strength of the proposed SOGNN is that it could automatically extract graph structure from EEG features. The graph structure of SOGNN is dynamic and independent for different EEG features. As a result, the proposed SOGNN obtained more accurate and robust emotion recognition performance. In the next section, we will discuss and analyze the proposed model.

## 4. Discussion

In this section, we analyze the proposed method and its internal properties in detail. We will discuss the performance differences of the SOGNN model with different features, self-organized graphs with different top-k rates, different graph construction methods, interchannel connections, etc.

**Figure 4:**
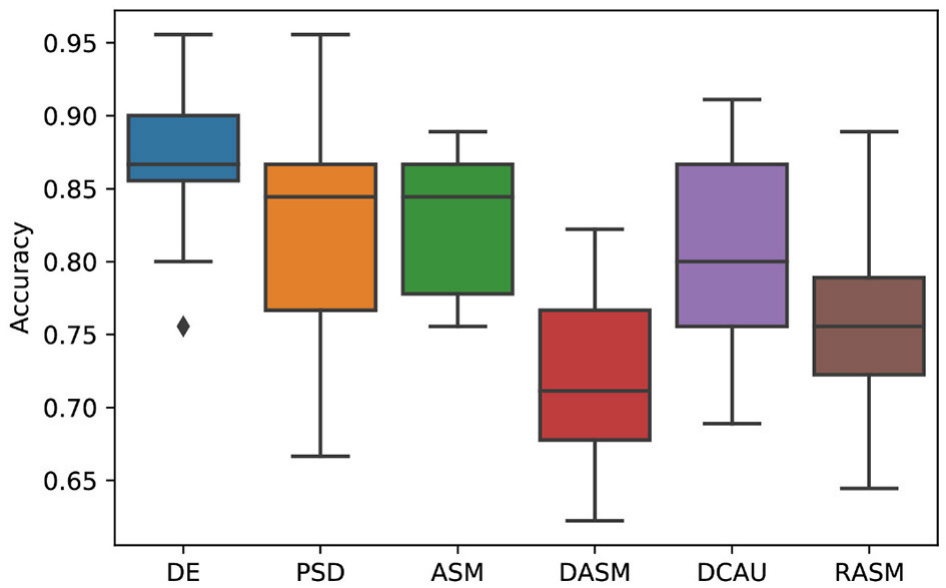
Emotion recognition performance of SOGNN with DE, PSD, ASM, DASM, DCAU, and RASM features.

Figure 4 shows the emotion recognition accuracies of the proposed SOGNN model with different features including DE, PSD, ASM, DASM, DCAU, and RASM features. We found that the DE feature is the most discriminate feature while the performances of the other features are much lower. This finding is consistent with previous researches (Song et al., 2019b; Zhong et al., 2020).

**Figure 5:**
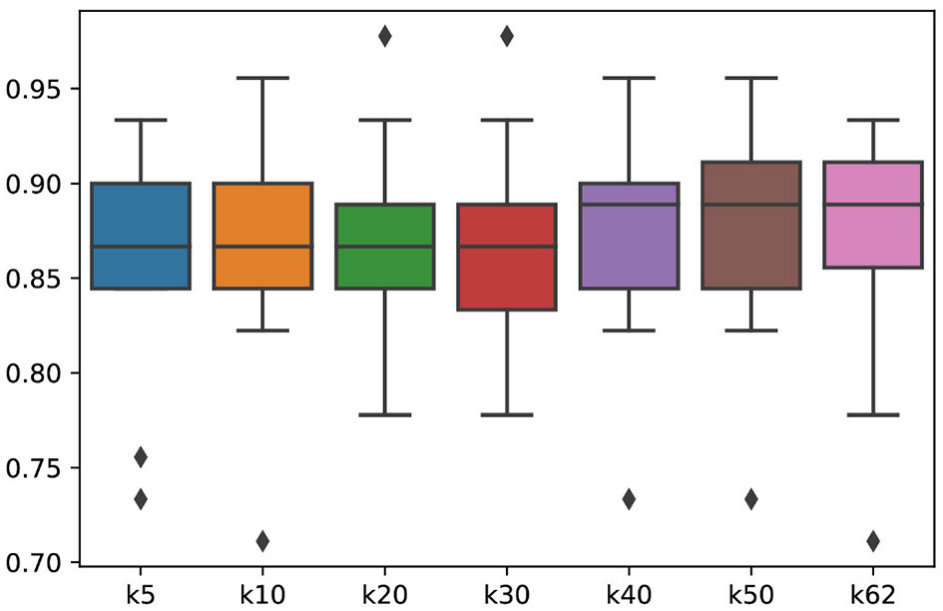
Performance changes of SOGNN as the variance of top-k sparse graph varies.

Accordingly, dense graph convolution usually has high computational costs. Therefore, it is significant to construct a sparse and effective graph in practice. To obtain a sparse adjacent matrix of graph, we applied the top-k technique in which only the k-largest connection weights of each EEG electrode in the adjacent matrix were maintained while the remaining small weights were set to zero. As shown in Figure 5, the performance of the SOGNN with different top-k sparse graphs is presented. In the figure, k-10 denotes that only the 10 largest connection weights were maintained while the remaining weights were set to zeros. Likewise, k-62 indicates that the total connections between all 62 electrodes were reserved. We can find that the model with k-10 connections achieved similar performances as those models with more connections. This finding indicates the effectiveness of the model with sparse adjacent matrix.

**Figure 6:**
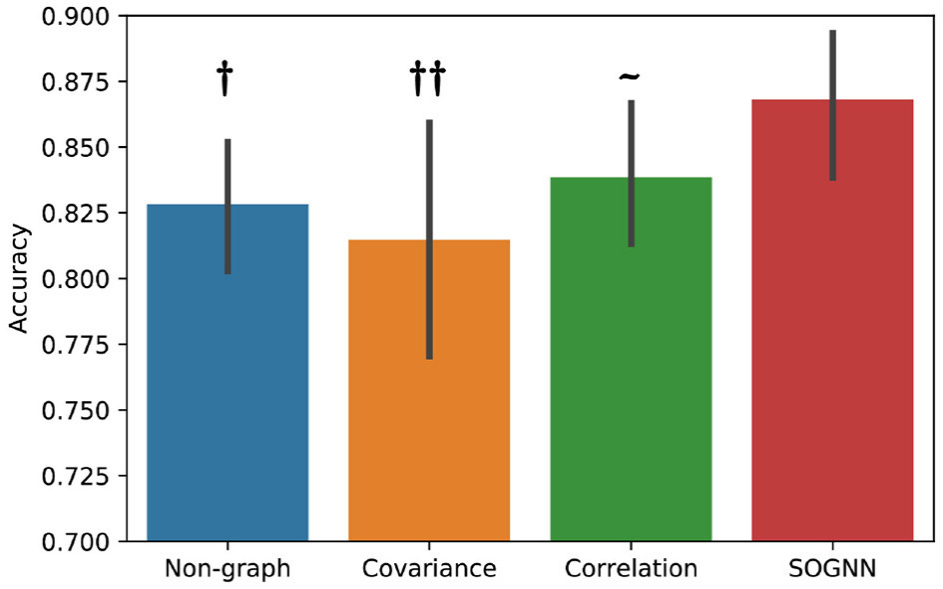
Emotion recognition performance based on different graphs. Wilcoxon signed rank test: ~ non-significant, †*p* < 0.05, ††*p* < 0.01.

In the proposed SOGNN model, a self-organized graph construction module is applied to dynamically learn the interchannel relationships of EEG signals across subjects. Here, we investigate different graph construction technique and their performance. Figure 6 presents the emotion recognition accuracies on SEED dataset of the models with different graphs. To compare the performance of the models with different graphs, we would like to conduct statistical analyses. Evaluated on a dataset with only 15 subjects, the results of each model may not follow normal distribution. Wilcoxon signed-rank test is a non-parametric statistical hypothesis test which is suitable for the analysis on non-normally distributed data. With Wilcoxon signed-rank test result, we are able to determine whether the proposed model could achieve statistically significant better performance than the other models. As shown in Figure 6, the SOGNN achieved significantly better performance than non-graph model and the model with covariance graph. Regarding the covariance graph, the values of the elements in its adjacent matrix are usually too large that the graph convolutional layers will be easily saturated. This might be the reason for the low performance of the model with the covariance graph. The correlation graph can be considered as a normalized version of the covariance graph in which its adjacent matrix is normalized to be in [0, 1]. As a result, its performance is improved a little. Here, we propose a straightforward method termed self-organized graph construction in (5). The proposed SOGNN could achieve state-of-the-art emotion recognition performance on the SEED and SEED-IV datasets. Our experiments demonstrated the effectiveness of the proposed model and the self-organized graph construction method.

**Figure 7:**
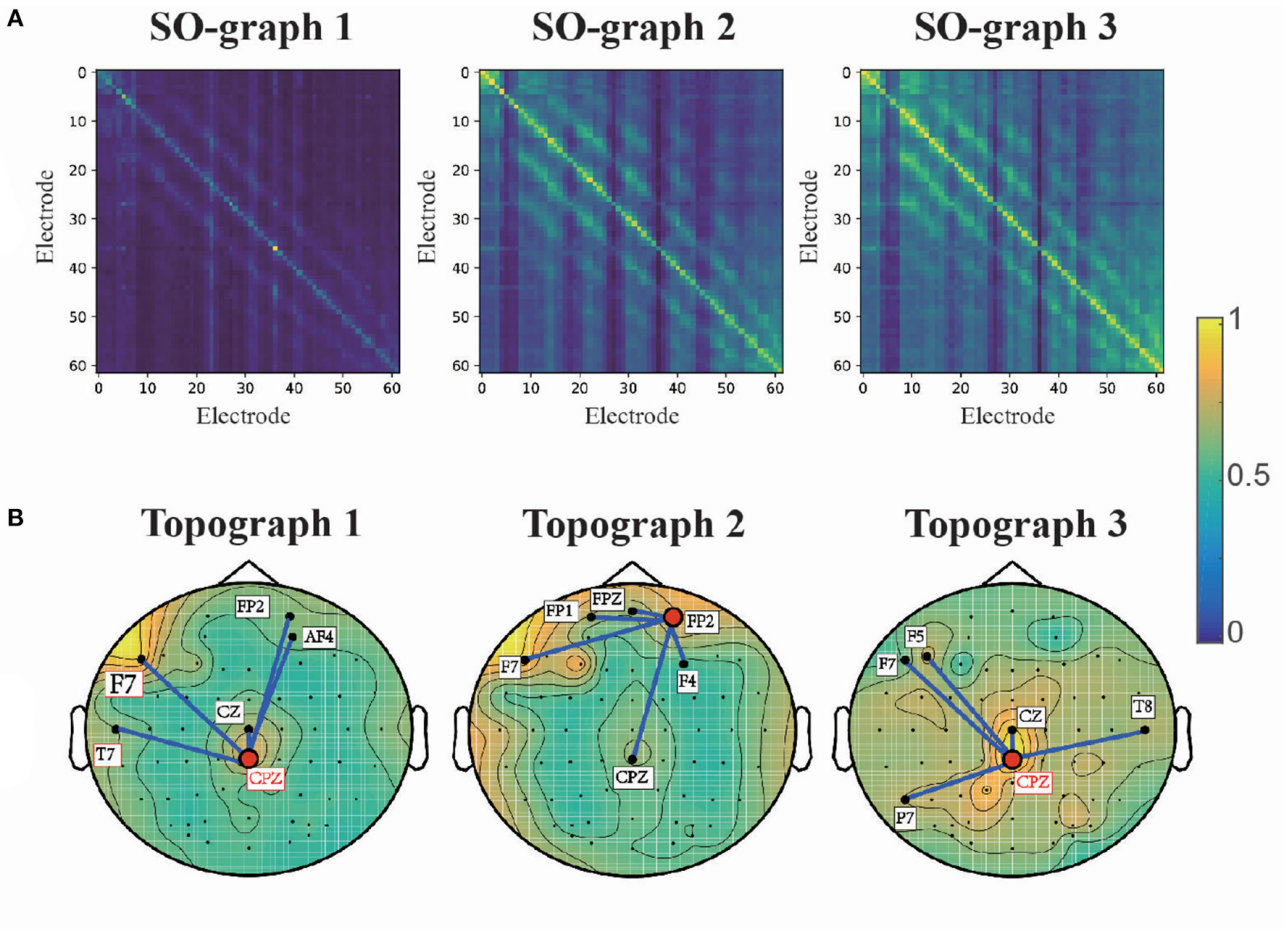
Adjacent matrixes **(A)** and topographic maps **(B)** learned by SOGNN.

To analyze the interchannel relationships learned by the proposed model, we obtained the average adjacent matrix of its self-organized graph (SO-graphs 1-3 as indicated in Figure 3) for SEED samples. Then, the average adjacent matrixes of SO-graphs 1-3 are normalized to [0, 1] for ease of analysis and presented in Figure 7A. These graphs reflect the common connections of EEG electrodes for emotion recognition. The SO-graph 1 is diagonally dominant that only few diagonal elements are relatively large while most of the rest elements are close to zero. That is only the features of a few EEG channels are discriminative for first graph convolution layer. Moreover, the off-diagonal elements of SO-graph 2 and 3 indicated that interchannel relationships also play important roles in classifying different emotion EEG signals.

Furthermore, we analyze the interchannel connections of the learned graphs for emotion recognition. We extracted the diagonal elements of the adjacent matrixes for SO-graph 1-3 and transformed into topographic maps. The topographic maps for SO-graphs 1-3 are presented in Figure 7B. According to the topographic maps, the prefrontal, and centro-parietal electrodes (e.g., F7, CPZ, FP2) had the largest weights in the topographic maps. The five electrodes with the largest weights connected with CPZ and FP2 are also presented. According to a previous study (Davidson et al., 1999), the activation in the regions of prefrontal cortex is related to blunted positive and negative emotions. A positive waveform will be enhanced over the centro-parietal electrode (CPZ) for emotional pictures (Lang and Bradley, 2010). In many related studies (Tyng et al., 2017; Alia-Klein et al., 2018; Pan et al., 2018), the prefrontal-parietal network is activated by emotion-related stimulus such as facial feelings, negative emotion processing, anger, etc. The interchannel relations between prefrontal, parietal and occipital channels are discriminative for emotion recognition EEG signals. Our findings coincide with the spatial distribution for emotion, as suggested by prior studies.

The above experiments and analysis of the proposed SOGNN model are significant for EEG-based emotion recognition. As a novel graph processing method for brain signals, it may bring some inspiration for neuroscience research, such as graph-based functional magnetic resonance imaging data processing.

## 5. Conclusion

In this paper, a novel model termed SOGNN was proposed for cross-subject emotion recognition. The SOGNN model was able to dynamically learn the interchannel relationships of EEG emotion signals using a self-organized graph construction module. The proposed model achieved state-of-the-art performance on two open EEG emotion recognition databases, i.e., SEED and SEED-IV. In addition, a series of analyses demonstrated the effectiveness of the proposed model on graph construction and emotion recognition. The experimental results indicated that the SOGNN model is not only an effective model for recognizing emotions, but it is also a potential technique for other EEG-based applications. In the future, we would like to build more efficient networks to model brain signals and effectively decode high-level cognitive behaviors. Moreover, some new emerging machine learning techniques can also inspire the methodology for emotion recognition and affective computing.

## Data Availability Statement

Publicly available datasets were analyzed in this study. This data can be found here: https://bcmi.sjtu.edu.cn/resource.html.

## Ethics Statement

The studies involving human participants were reviewed and approved by Ethics Committee of South China Normal University. The patients/participants provided their written informed consent to participate in this study. Written informed consent was obtained from the individual(s) for the publication of any potentially identifiable images or data included in this article.

## Author Contributions

JL proposed the idea, conducted the experiments, and wrote the manuscript. SL and FW provided advice on the research approaches, signal processing, and checked and revised the manuscript. JP offered important help that guided the experiments and analysis methods. All authors contributed to the article and approved the submitted version.

## Conflict of Interest

The authors declare that the research was conducted in the absence of any commercial or financial relationships that could be construed as a potential conflict of interest.
